# An extrahippocampal projection from the dentate gyrus to the olfactory tubercle

**DOI:** 10.1186/1471-2202-6-38

**Published:** 2005-05-31

**Authors:** Heinz Künzle

**Affiliations:** 1 Institute of Anatomy, Ludwig Maximilians-University, Pettenkoferstrasse 11, D-80336 Munich, Germany

## Abstract

**Background:**

The dentate gyrus is well known for its mossy fiber projection to the hippocampal field 3 (CA3) and its extensive associational and commissural connections. The dentate gyrus, on the other hand, has only few projections to the CA1 and the subiculum, and none have clearly been shown to extrahippocampal target regions.

**Results:**

Using anterograde and retrograde tracer techniques in the Madagascan lesser hedgehog tenrec (Afrosoricidae, Afrotheria) it was shown in this study that the dentate hilar region gave rise to a faint, but distinct, bilateral projection to the most rostromedial portion of the olfactory tubercle, particularly its molecular layer. Unlike the CA1 and the subiculum the dentate gyrus did not project to the accumbens nucleus. A control injection into the medial septum-diagonal band complex also retrogradely labeled cells in the dentate hilus, but these neurons were found immediately adjacent to the heavily labeled CA3, while the tracer injections into the rostromedial tubercle did not reveal any labeling in CA3.

**Conclusion:**

The dentate hilar neurons projecting to the olfactory tubercle cannot be considered displaced cells of CA3 but represent true dentato-tubercular projection neurons. This projection supplements the subiculo-tubercular projection. Both terminal fields overlap among one another as well as with the fiber terminations arising in the anteromedial frontal cortex. The rostromedial olfactory tubercle might represent a distinct ventral striatal target area worth investigating in studies of the parallel processing of cortico-limbic information in tenrec as well as in cat and monkey.

## Background

The dentate gyrus (Dt) may be known best for its input from the entorhinal cortex and its mossy fiber projection to the CA3 [1-3]. In addition, the Dt has extensive associational and commissural connections [4-8] considered crucial for the generation of gamma frequency oscillations and learning (e.g. [9-11]). Most attention among the dentate interneurons may recently have received the cells in the hilus region (DtHi) due to their vulnerability to various injuries [12-14] as well as their remarkable structural, histochemical and electrophysiological diversity [15-20]. A few hilar neurons also project to the cornu ammonis (CA) and the subiculum (Sbi) [15,21]. Extrahippocampal projections from the dentate gyrus, however, are not known with the exception from some septal projecting neurons in the DtHi [22-25] usually considered displaced cells of CA3 [1,21].

Continuing our attempts to elucidate the hippocampal and parahippocampal circuits in mammals with a poorly differentiated brain [26-29] the present study in the lesser hedgehog tenrec (*Echinops telfairi*, *Et*) will demonstrate some true dentate hilar cells projecting to a circumscribed region in the olfactory tubercle. The projection may represent an additional parallel pathway transferring cortico-limbic information to the ventral striatum [30-32].

## Results

### Axo-terminal labeling in the striatum following tracer injections into the dentate gyrus

**Figure 1:**
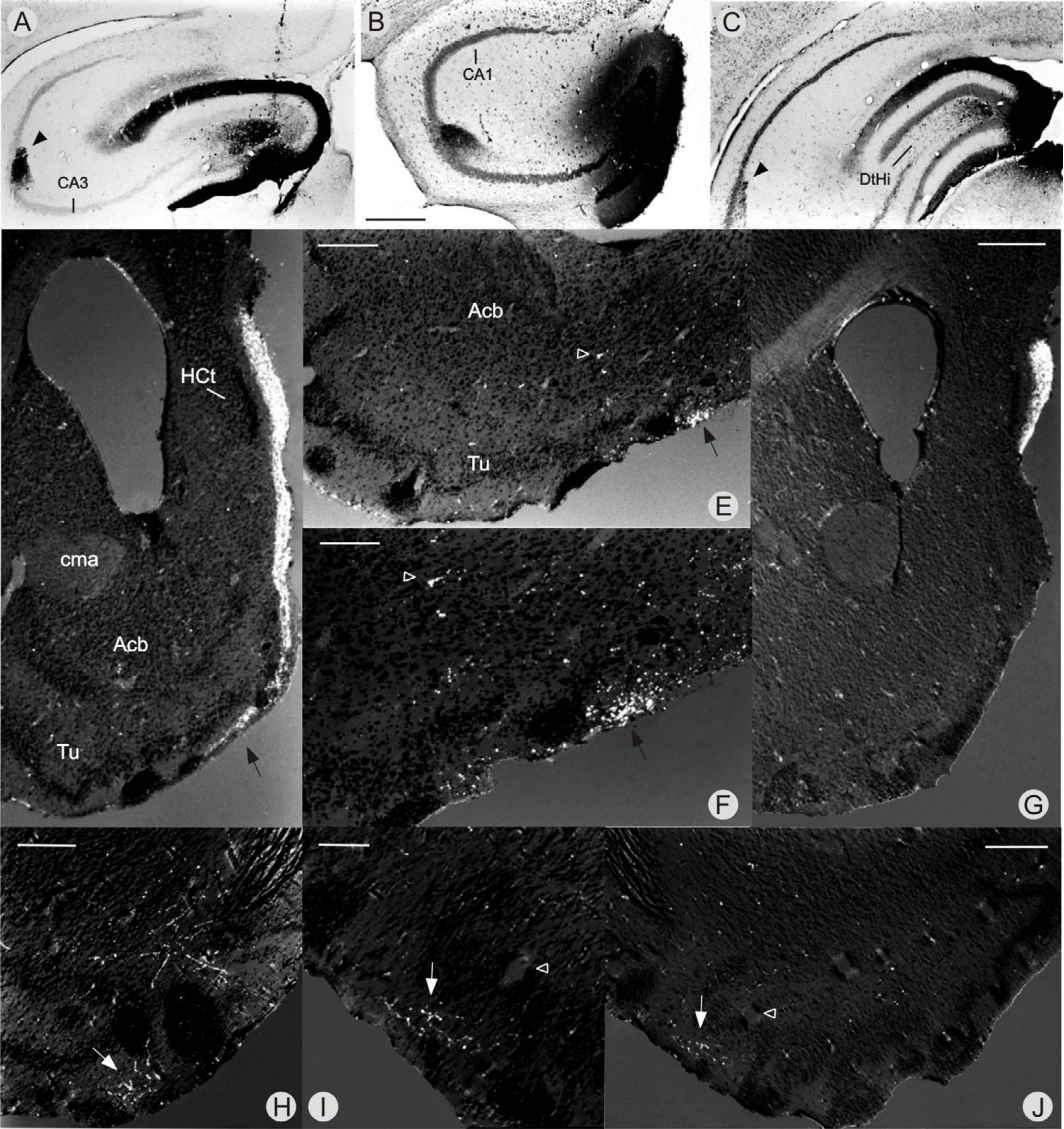
**Anterograde labeling in the olfactory tubercle following tracer injections into the dentate gyrus. **Photomicrographs taken by transmitted and polarized light showing anterograde projections to the olfactory tubercle (D, E, F, H, I, J; arrows point to labeling) and/or the anterior hippocampal continuation (D, G) following tracer injections into the dentate gyrus in Et01-21B (A, D), Et98-49W (B, E, F), Et01-36B (C, G) and Et00-11B (H – J). The molecular layer of Tu was labeled in all cases (D-H, ipsilateral side; I and J, contralateral side) but Et01-36B (G) where the tracer injection scarcely involved the DtHi (note the low labeling density of the mossy fiber projection to CA3 in C [arrow head] as compared to A). The Acb (D, E) was not labeled following tracer injections into Dt unlike the cases injected with tracer into CA1 / Sbi. All BDA cases also clearly labeled the HCt (D, G) due to the retrograde-anterograde collateral transport of the tracer within the entorhino-hippocampal system described previously [28]. Open arrow heads in E, F and I, J respectively point to similar location. Scale bars in B (also applying to A and C) and G (also applying to D) 0.5 mm; in E and J = 0.4 mm, in H = 0.3 mm; in F and I = 0.2 mm.

Among the experiments with tracer injections into the hippocampus (Table 1) two cases were injected with biotinylated dextran amine (BDA) into the dentate area exclusively. These tracer injections mainly involved the dentate molecular layer (DtMo) but there was some tracer uptake by the granule cells as seen from the weak to moderate mossy fiber projection to CA3 (Fig. 1). The sparse (Et01-36B, Fig. 1C) to moderate (Et01-21B, Fig. 1A) number of labeled cells in the dentate hilar region subjacent to the main injection site might be due to a retrogarde transport and/or a direct uptake of tracer substance. In Et00-11B and Et98-49W (Fig. 1B) all layers of Dt as well as the adjacent portions of CA3 were labeled directly with BDA and wheat germ agglutinin conjugated to horseradish peroxidase (WGA-HRP), respectively (the letters B and W following the case number indicate the tracer injected). The Dt and portions of CA1 were injected with BDA in Et00-08B, while in the remaining experiments the injection involved the subiculum (Sbi) with (Et00-13W) or without (Et00-41B) an involvement of the Dt and CA1.

The latter two cases revealed the well-known hippocampo-striatal projection pattern to the nucleus accumbens (Acb) and the olfactory tubercle (Tu) [29]. No hippocampo-striatal projection, on the other hand, was found in Et01-36B (Table 1, Fig. 1G) injected with tracer almost exclusively into the DtMo. The remaining cases consistently showed some fiber labeling in the Tu (Fig. 1E,1F,1H) but did not reveal any projections to the Acb (Table 1; Fig. 1D). The labeling in Tu involved especially the molecular layer in the medial quarter of Tu at levels rostral to the insula magna. In all cases but Et01-21B the hippocampo-tubercular projection was represented on both sides with a slight ipsilateral predominance (Fig. 1H,1I,1J).

In Et01-21B the course of fibers towards Tu appeared to pass exclusively within the molecular layer of the hippocampal continuation (HCt; Fig. 1D; also note the difference to Fig. 1G of Et01-36B). In the other cases labeled fibers were seen in the HCt as well as the fornix. A fiber course across the fornix appeared most likely for the contralateral projection as far as the contralateral HCt was almost unlabeled and a fiber crossing was never observed for the HCt-fibers unlike the fornix fibers.

For the subsequent experiments carried out in order to identify more accurately the origin of the hippocampo-tubercular projections, it should be mentioned that similar to other reports [33] there were also hippocampal projections to the diagonal band-medial septum complex (DgMS). Evidence for these projections was obtained from the BDA experiments which revealed an extensive terminal-like fiber pattern across the DgMS, but only few, if any labeled perikarya unlike the WGA-HRP experiments (Table 1).

### Perikaryal labeling in the dentate gyrus following tracer injections into the striatum and septum

**Figure 2:**
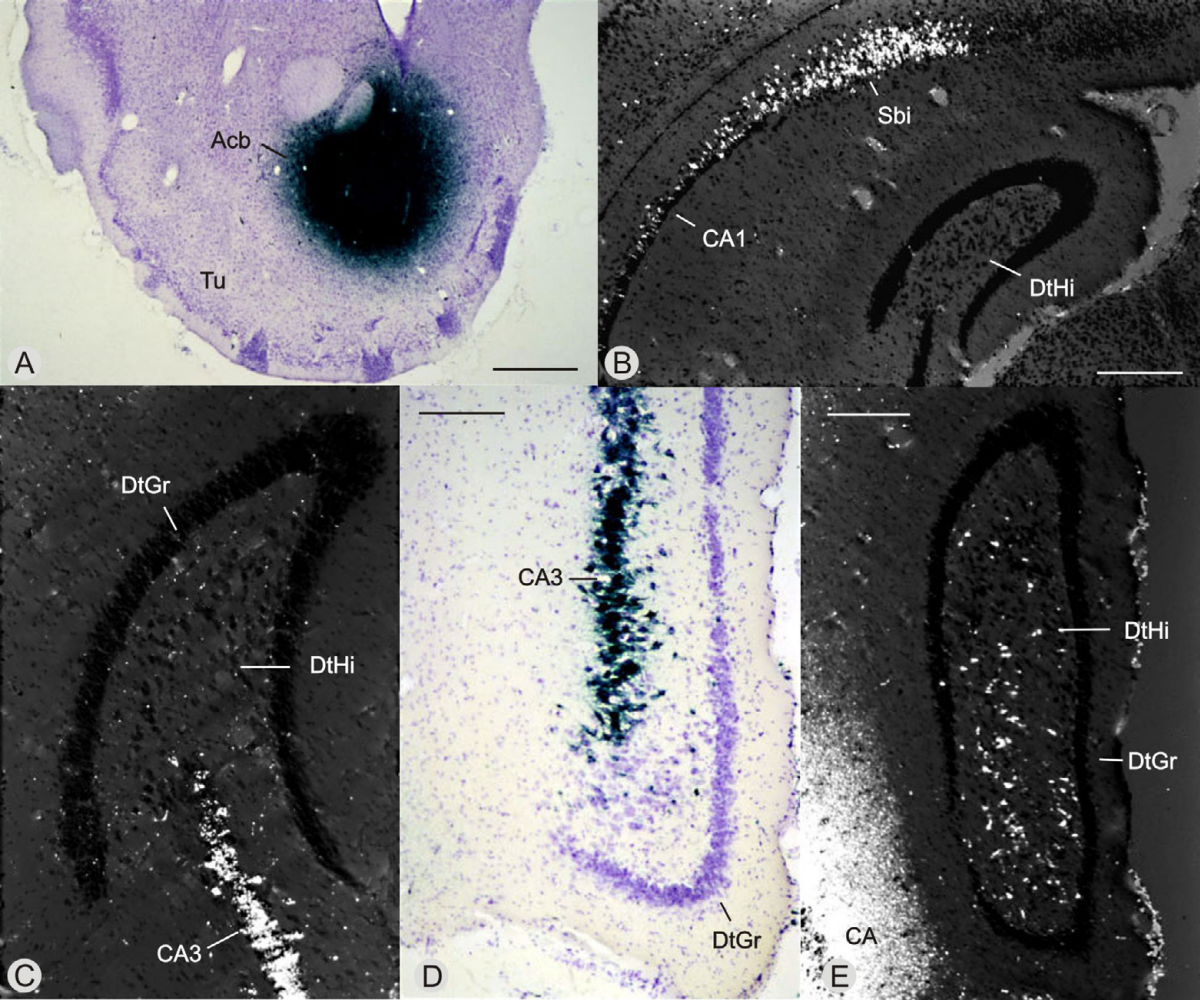
**Retrograde labeling in the hippocampus following tracer injections into nucleus accumbens and septum. **Following WGA-HRP injection into the Acb (A) in Et02-65W there were numerous retrogradely labeled cells in Sbi and CA1 (B), but none in CA3 and Dt (B). The tracer injection into the septum of Et00-08W (see Fig. 10L in [26]) also labeled heavily the CA3 (C-E) as well as some cells in the adjacent DtHi (D, E), particularly at caudal levels (section D is taken 960 μm caudal to C and 160 μm rostral to E). Scale bars = 0.8 mm in A, 0.4 mm in B, 0.3 mm in E and 0.2 mm in D (also applies to C).

**Figure 3:**
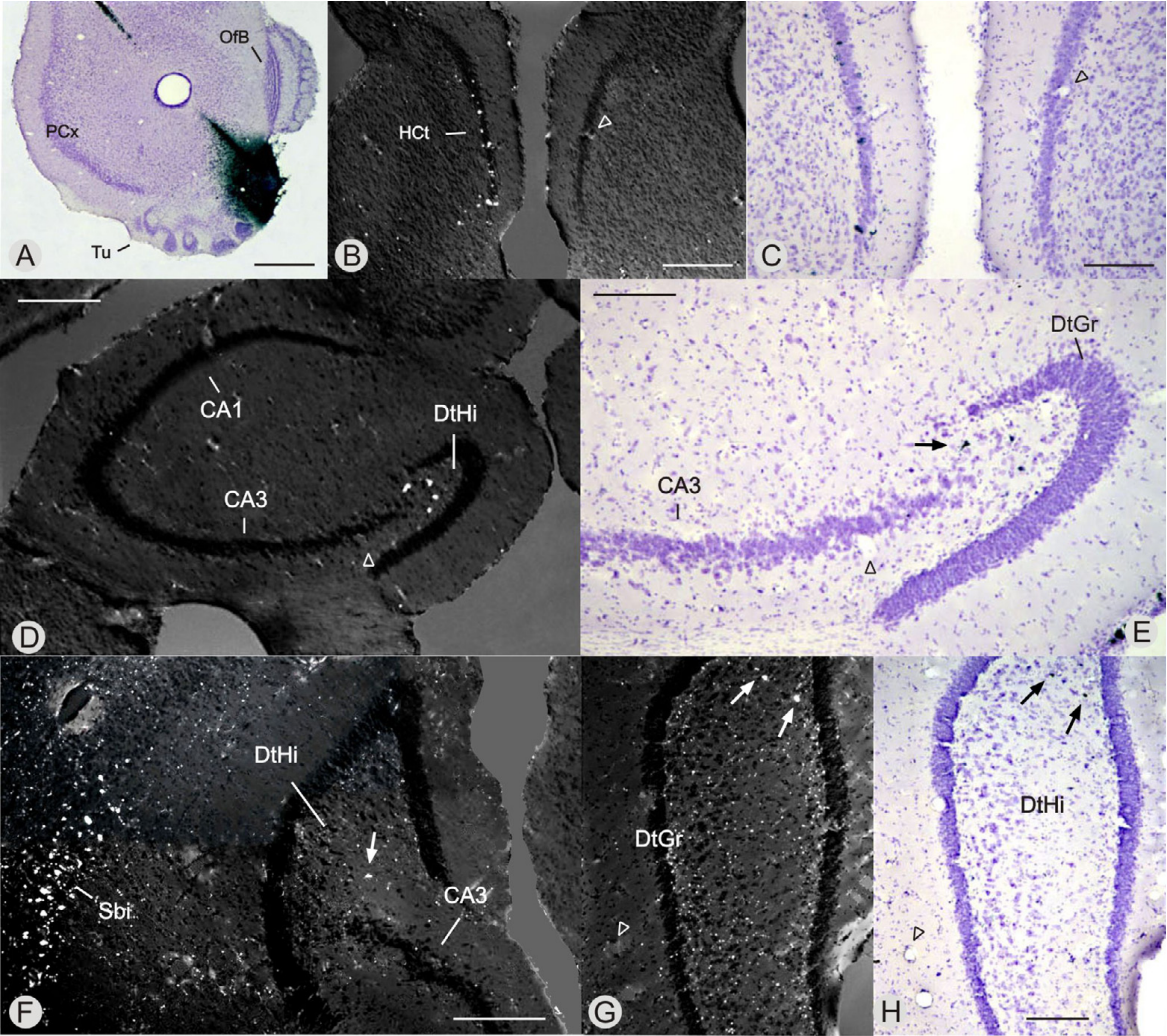
**Retrograde labeling in DtHi and HCt following tracer injections into the olfactory tubercle. **Tracer injections into the rostromedial Tu (A) consistently labeled a few dentate hilar neurons (D-H; arrows point to labeled cells), but failed to label the CA3 (D-F). A-E are from Et03-58W, F-H from Et01-47W. Some labeled neurons are also noted in the ipsilateral, anterior HCt (B, C) confirming previous anterograde data [28]. Remarkably, both the HCt and the DtHi project to the rostromedial Tu, but not to the Acb. Arrow heads point to similar location. OfB, olfactory bulb; PCx, paleocortex. Scale bars = 0.8 mm in A, 0.4 mm in B and D, 0.3 mm in F, 0.2 mm in C, E and H (as G).

There were two cases with WGA-HRP injections involving the rostromedial Tu including its molecular layer (Table 2). The tracer injection in Et03-58W showed a relatively circumscribed injection site (Fig. 3A); a direct labeling of the DgMS, however, could not be excluded. In Et01-47W there was some loss of tracer along the electrode tract leading to a contamination of the third layer of the HCt. A control injection into the ventral septum including the DgMS (Fig. 10L in [26]) was available too as well as two tracer injections involving the Acb (Table 2; Fig. 2A).

Consistent with previous studies in subprimate species [34-38] the tracer injections into the Acb led to retrograde labeling of exclusively the Sbi and CA1 (Fig. 2B; regarding the involvement of CA3 in monkey see ref.[39]). The septal injection, in addition, labeled numerous neurons in CA3 (Fig. 2C, 2D) as well as a substantial number of cells in the DtHi adjacent to the heavily labeled CA3, particularly at caudal levels (Fig. 2D, 2E). In the cases injected with tracer into the Tu labeled cells were found in the HCt (Fig. 3B, 3C), the Sbi (Fig. 3F) and the DtHi (Figs. 3D,3E,3F,3G,3H). These cases, however, consistently failed to show any retrograde labeling in the CA3 (or the dentate granule cells layer). In Et03-58W the labeled neurons in the DtHi were restricted to its rostral third, while in Et01-47W they involved rostral as well as caudal portions of the hilus. The latter case also showed a very few labeled cells on the contralateral side. A particular distribution of the labeled cells within the DtHi was not obvious, and their poor dendritic labeling did not allow a further characterization of the cells.

## Discussion

Unlike the dentate projection to the HCt the projection to the olfactory tubercle cannot be explained by a retrograde-anterograde collateral transport of tracer [28]. Tubercular terminations are not seen in the case with a BDA injection largely confined to the dentate molecular layer (Fig. 1C,1G), and tubercular projections are noted in the cases with dentate injections of WGA-HRP (Fig. 1B,1E,1F), a tracer substance not known to be transported in a retrograde-anterograde collateral fashion [40]. Moreover, the dentate-hilar neurons are also filled by the retrograde axonal flow from the tracer deposits in the Tu.

The projection to the olfactory tubercle differs from previously reported extrahippocampal projections from the dentate area as far as its cells of origin are confined to the DtHi while the septal [22,23,41] and hypothalamic [42] projections arise predominantly from the CA and the Sbi, only sparsely from the DtHi. Unlike the latter cells, therefore, the dentato-tubercular neurons can not be considered displaced cells of CA3. We have not characterized yet the particular morphology and histochemistry of these neurons [16,26,43-45] and do not know whether their projections are distinct ones or arise as collaterals [46-49]. Nevertheless, it is a projection which involves a circumscribed region in the olfactory tubercle and avoids the nucleus accumbens, the main striatal target area of the hippocampus [34,35,39].

These findings are in line with previous suggestions considering the medial Tu as a separate striopallidal subdivision [50-53] and fit with the concept of the parallel processing of cortico-striatal information [30,31,54-56]. The question remains as to why a dentato-tubercular projection has not been demonstrated so far in other species. The projection may be unique to tenrecs or mammals with little differentiated brain but its apparent absence in other mammals may as well be explained by the low strength of its projection and/or its circumscribed field of termination. Such a projection may easily be overlooked in anterograde tracer studies, especially in species with a large brain. Retrograde tracer studies, on the other hand, have only been done in the rat and the hamster [37,50,57,58] and it is questionable, whether their small circumscribed dentato-tubercular target area, if present, has been injected with tracer. Rodent species, in addition, are not representative for studying the hippocampo-tubercular projections. In the rat, the Tu receives considerably less hippocampal afferents from the CA1/Sbi fields [35] than in the tenrec [29], cat [34] and monkey [39]. Similarly, the relative strength of the entorhinal projections to the Tu as compared to the Acb is much less in rodents [36,59,60] than in non-rodent species [29,39,61]. It may also be noted that the hippocampal continuation, the only other striatal input region projecting exclusively to the tenrec's rostromedial Tu [28], is poorly represented in the rat compared to other mammals [62]. One may speculate that the dentato-tubercular neurons in the DtHi represent an extension of the HCt into the dentate gyrus and are only present in species showing a well differentiated HCt as e.g. in tenrecs and primates [62].

Unfortunately we still know little about the precise connectivity of the circumscribed dentato-tubercular target area. It receives few if any direct projections from the olfactory bulb (personal reinvestigation of the material published previously [63]) but gets a distinct input from the anteromedial frontal cortex [29]. The dentato-tubercular target area is likely to be part of a cortico-basal ganglia-thalamo-cortical loop involving the mediodorsal nucleus [64-67] and the prefrontal cortex, at least in subprimate species (rat: [68-70]; cat: [71]; monkey: [72-74]). Notably, while the prefrontal and hippocampal afferents to the Acb terminate in a predominantly non-overlapping fashion [29,32,75], the cortico-striatal fibers appear to overlap considerably with the hippocampal afferents in the Tu.

## Conclusion

The present data show for the first time a projection from the dentate gyrus to the rostromedial olfactory tubercle. Its cells of orgin, located in the dentate hilus, cannot be considered displaced neurons of CA3 but represent true dentato-tubercular projection neurons. The projection is assumed to be present in other mammals, too. In the tenrec the dentato-tubercular projection overlaps with the subiculo-tubercular projection and the fiber terminations arising in the anteromedial frontal cortex. The circumscribed ventral striatal target area appears worth investigating in studies of the parallel processing of cortico-limbic information in tenrec as well as in cat and monkey.

## Methods

### Animals

The lesser hedgehog tenrec (*Echinops telfairi*, Et) is a member of the tenrecomorpha classically considered an insectivoran suborder [76] but actually grouped within the superorder Afrotheria (order Afrosoricida e.g. [77,78]). The animals were obtained from our breeding colony [79] built up to investigate their little differentiated forebrain and get an insight into the evolution of the mammalian brain [80,81].

### Tracer injections

Eleven tenrecs weighing between 80 and 140 gm were anesthetized with tribromoethanol (1.0 ml/100 gm, i.p.) and injected with tracer into the hippocampus (n = 7), the ventral striatum (n = 4) and the septum (n = 1) following the German laws on protection of animals (one animal received two injections at different locations). Wheat germ agglutinin conjugated to horseradish peroxidase (WGA-HRP; Sigma; n = 7) and biotinylated dextran amine (BDA; Sigma; n = 5) were used as tracer substances. Most cases have been described previously with regard to other connections [26,28,82]. The additional tracer injections (Et01-47W, Et03-58W) were done in the same fashion: The WGA-HRP (1.5–8 nl of a 2–5% solution in distilled water) was pressure injected through a glass micropipette (tip diameter 8–15 μm) attached to a Hamilton syringe driven by a micromanipulator. The BDA was injected iontophoretically (10 % in 0.01 M phosphate buffer, pH 7.25; current of 2–5 μA for 5–10 min). After a survival time of 2–7 days the animals were reanesthetized and perfused transcardially with saline. The fixation consisted of a phosphate-buffered solution containing 1% paraformaldehyde and 2.5% glutaraldehyde, followed by a phosphate-buffered solution containing 15% sucrose.

### Tissue processing

The brains were soaked overnight in a 30% phosphate-buffered sucrose solution, embedded in an albumin-gelatine mixture and cut on a freezing microtome at 40 μm in the frontal plane. The WGA-HRP staining was usually done on two out of four sections according to the standard tetramethylbenzidine technique [83] and a modified tungstate-stabilized tetramethylbenzidine technique [84]. Following a pretreatment in mercaptoethanol (2% in 0.05 M PBS at 37° for 5–15 min in order to suppress a staining of WGA-HRP) the BDA was visualized by incubating the sections in extravidin-peroxidase and developing the reaction product with nickel-enhanced diaminobenzidine and tungstate-stabilized tetramethylbenzidine (modified according to [84]) in an alternating fashion. The mounted sections were counterstained with neutral red (following treatment with diaminobenzidine and tetramethylbenzidine) and cresyl violet (following use of tungstate-stabilized tetramethylbenzidine). The tungstate-stabilized tetramethylbenzidine proved quite useful for visualizing faint projections but might result in large cristalline artefacts, particularly after overnight incubation.

### Data analysis

Bright-field illumination and polarized light were used for the analysis. Images were captured on an Ilford 50 negative film or a Fujichrome 64T positive film (using a Zeiss axiophot microscope), scanned (Nikon Coolscan 5000) and transported into Adobe Photoshop (v. 7.0) and Corel Draw (v. 11.0). The sharpness, contrast and brightness were adjusted to reflect the appearance of the labeling seen through the microscope. For the sake of clarity the most disturbing crystalline artefacts were removed, particularly in darkfield images. In some darkfield micrographs the light reflecting embedding medium was also removed. Furthermore to facilitate the analysis all micrographs are shown with the injection site on the left.

## List of Abbreviations

Acb – nucleus accumbens

BDA – biotinylated dextran amine

CA1/3 – subfields of cornu ammonis

cma – commissura anterior

DgMS – diagonal band-medial septum complex

Dt – dentate area

DtGr – dentate granule cell layer

DtHi – dentate hilar region

DtMo – dentate molecular layer

Et – Echinops telfairi

HCt – hippocampal continuation

Sbi – subiculum

Tu – olfactory tubercle

WGA-HRP – wheat germ agglutinin conjugated to horseradish peroxidase

## Figures and Tables

**Table 1:** Cases showing hippocampo-basal telencephalic projections with anterograde tracing techniques

**Case**	**Injection site**	**Terminal projections**		
		
		**Tu**	**Acb**	**DgMS**
Et01-36B	DtMo>>DtGr_±DtHi_	-	-	-
Et01-21B	DtMo>DtGr_±DtHi_	**++**	-	±
				
Et00-11B	Dt>CA3	+	-	**+++ **^b^
Et98-49W	Dt>CA3	+	-	**++ **^c^
				
Et00-08B	Dt, CA1	**+**	-	**++ **^a^
Et00-13W	Dt, CA1<Sbi	**+**	**++**	**+++ **^c^
Et00-41B	Sbi	**++**	**++**	**+++ **^a^

Letters B and W following the case numbers indicate the tracer injected, BDA and WGA-HRP respectively. The regions injected with tracer are written with large or small letters depending on their major or minor contribution to the projection pattern (± indicates questionable involvement). The symbols -, ±, **+**, **++**, **+++ **(none, questionable, weak, moderate, strong) refer to the overall intensity and extent of the projections. The letters superscripted in column DgMS refer to additional labeling of isolated (a), few (b) and numerous (c) cell bodies.

**Table 2:** Cases showing hippocampo-basal telencephalic projections with retrograde tracing techniques

**Case**	**Injection site**	**Origin of projection**			
		
		**DtHi**	**CA3**	**CA1**	**Sbi**
Et03-58W	TuM	**+**	-	-	±
Et01-47W	TuM_+HCt_*	+±	-	±	**++**
					
Et02-63W	Acb_+FrCx_	-	-	**++**	**+++**
Et02-65W	Acb	-	-	**++**	**+++**
					
Et00-08W	SeV	**++**	**+++**	**+++**	**+++**

The areas injected with tracer and the density of labeled perikarya are judged as in Table 1.

* The contamination of the anterior HCt by the tracer is restricted to the third layer not connected with the hippocampus [28]. FrCx, frontal cortex; SeV, ventral septum.
